# Is the bark worse than the bite? Additional conditions used within community treatment orders

**DOI:** 10.1192/pb.bp.113.043422

**Published:** 2014-02

**Authors:** Mike Smith, Tim Branton, Alastair Cardno

**Affiliations:** 1 Partnerships in Care; 2 Leeds and York Partnership NHS Foundation Trust; 3 University of Leeds

## Abstract

**Aims and method** To investigate the use of additional conditions attached to community treatment orders (CTOs) and whether they influence the process of recall to hospital. We conducted a retrospective descriptive survey of the records and associated paperwork of all the CTOs started in the trust in the year from January 2010. Each CTO was followed up for 12 months.

**Results** A total of 65 CTOs were included in the study; 25 patients were recalled during the study and all but one of these had their CTO revoked and remained in hospital. Each patient whose CTO was revoked had experienced a relapse in their condition. Many patients had not complied with CTO conditions prior to relapsing and could potentially have been recalled earlier.

**Clinical implications** Our findings suggest that the breaching of additional CTO conditions does not tend to result in a patient's recall to hospital. This has implications regarding how the workings of CTOs are explained to patients and regarding the utility of additional conditions more generally.

Community treatment orders (CTOs) were introduced in England in 2008. They were intended to be used to prevent patients with severe and enduring mental health problems deteriorating in the community and were specifically targeted at ‘revolving-door’ patients.^1^ The proposed legislation was controversial. Two large reviews^2,3^ failed to find an evidence base to support the claims made for compulsory community treatment. One review did, however, acknowledge the difficulties in conducting studies in this area.^3^ The main criticisms of CTOs relate to the restrictions that they impose on patients' human rights.^4^

According to the Mental Health Act 1983, a CTO is only an option for patients who meet the following criteria:

- the patient has a mental disorder of a nature or degree which makes it appropriate for them to receive medical treatment
- it is necessary for the patient's health or safety or for the protection of others that the patient should receive such treatment
- subject to the patient being liable to be recalled, such treatment can be provided without the patient continuing to be detained in a hospital
- it is necessary that the responsible clinician should be able to exercise the power under Section 17E(1) of the Act to recall the patient to hospital
- appropriate medical treatment is available for the patient.

The Mental Health Act also stipulates that the CTO must include the conditions with which the patient is required to comply. Two conditions are mandatory. One requires patients to make themselves available for medical examination for consideration of extension of the CTO, and the other to allow a second opinion appointed doctor to conduct a review of treatment in patients who cannot or do not consent to treatment.

Additional conditions can also be included which are necessary or appropriate to:

- ensure that the patient receives medical treatment for mental disorder, or
- prevent a risk of harm to the patient's health or safety, or
- protect other people.

Community treatment orders are akin to a contract to which the patient agrees so that they can be allowed to return to the community from hospital. The responsible clinician in charge of the CTO may attach any number of additional conditions to which the patient agrees to adhere. These additional conditions pertain to key areas of the patient's care such as adherence to medication and abstinence from drugs and alcohol.

Should the patient break this agreement and as a result pose an increased risk or begin to show early signs of relapse, their responsible clinician can recall them to hospital. One of the main aims of CTOs is to intervene early and prevent a patient relapsing.

## Method

### Data collection

A retrospective case-note review was conducted on all patients started on a CTO within Leeds and York Partnership NHS Foundation Trust between January 2010 and January 2011. Patients were only excluded from the final sample if their case notes could not be obtained. The study was granted approval as a service evaluation by the West Yorkshire Mental Health and Learning Disabilities Research Partnership in March 2011.

This sampling window of 12 months corresponded with 70 new CTOs. Data were collected for each of these CTOs for 12 months. Where CTOs ended before 1 year (due to discharge, revocation, etc.) the length of time for which the patient remained on the CTO was recorded.

In addition to demographic data, we collected information on:

- service involved (e.g. forensic, general adult)
- CTO outcome (renewed, recalled, revoked, discharged, lapsed) along with dates
- stated conditions included
- diagnosis
- evidence of previous non-adherence
- evidence of previous substance misuse
- evidence of whether CTO conditions were reviewed or varied
- episodes of informal admissions
- evidence of monitoring, breaches, and consequences of breaches of conditions.

## Results

### Patients in the sample

Medical records were available for 65 of the initial 70 CTOs. These 65 CTO episodes corresponded to a total of 58 patients (7 patients had two separate CTO episodes during the study period). We decided to include these 7 CTO episodes as when reviewed they were notably different from the other CTO episode involving the same patient, both with regard to the conditions included and the outcome.

The majority of CTOs were for patients who were male (66%), aged between 30 and 35 years (mean 44, s.d. = 12.8, range 23–83), had a psychotic illness (98%), were White British (68%), had a history of non-adherence to antipsychotic medication (98%), were being treated by the assertive outreach team (38%), and were in independent accommodation (82%). The majority of patients (63%) did not have a history of substance misuse. The use of depot medication was common, with 65% of patients on CTOs receiving antipsychotics via this route.

### Characteristics of the CTOs

All but one CTO (*n* = 64, 98%) originated from Section 3 of the Mental Health Act; the remaining CTOs originated from Section 37.

### Outcome of CTOs

Thirty-three CTOs (51%) were renewed and lasted for the duration of the study; 5 patients (8%) were discharged and 3 (4%) lapsed; 25 patients were recalled during the study period, and 24 of them had their CTO revoked.

### Conditions applied

A total of 241 additional conditions (not including mandatory conditions) were included across the 65 CTOs in the study. The mean number of conditions per CTO was 3.7 (s.d. = 3.14, range 1–8) and the modal value was 3.

The additional conditions were grouped into six categories (Table 1). There was some overlap between conditions, for example separate conditions specifying that the patient must comply with oral and depot medication. This overlap reduced the total number of additional conditions from 241 to 179 category-specific conditions. The conditions of access included conditions requiring patients to make themselves available for examination either through home visits or clinic appointments, the conditions of residence included conditions whereby a patient was instructed to reside at a particular location, and the conditions of drugs and alcohol included conditions whereby a patient had been instructed to abstain from drugs and alcohol and conditions that required the patient to make themselves available for drug testing.

Of the 11 miscellaneous conditions, 4 specified that the patient must accept help with their finances (accounting for 6 breaches), and 3 required the patient to engage with other professionals (e.g. probation), accounting for a further 2 breaches. One attached a requirement to engage in one meaningful activity daily and resulted in three breaches.

### Monitoring of conditions

The extent to which conditions were monitored varied between categories. All patients on depot medication had monitoring recorded; of those on oral medication, 82% had some evidence of monitoring taking place, commonly relying on patients' self-reports. In every case where a condition of access was stated, there was evidence that the clinical team made attempts to engage the patient and arranged out-patient appointments. Those with a condition

**Table 1:** Frequency and outcome of additional CTO conditions

Condition	Frequency *n*	Breaches *n*	Recalls *n*(%)^a^
Medication	65	43	4 (9)
Access	64	22	3 (14)
Residence	19	2	0
Drugs and alcohol	15	17	1 (0.6)
Medication monitoring	5	0	0
Miscellaneous	11	11	0
Total	179	95	8 (8)

CTO, community treatment order.

a. The recalls relate only to those which were associated with the breach of a condition. The actual number of recalls in the study was 24.

of residence were monitored only if they were in supported accommodation. Of the 15 patients with a condition of drugs and alcohol, only 7 had evidence of monitoring taking place.

### Breaches of conditions

There were 95 breaches across the 179 conditions (Table 1). The largest number of breaches occurred in the medication condition, followed by the condition of access, then the condition of drugs and alcohol. The drugs and alcohol category had the largest proportion of breaches (17 breaches out of 15 conditions).

Only 8 patients were recalled following a breach of one of their conditions, and 7 of them had relapsed by the time they returned to hospital. A further two patients were admitted informally. In the majority of cases a breach led to the patient being reminded that they were expected to comply with the conditions.

## Discussion

### Limitations

The main limitations relate to the measurement and recording of the data. The data collection method was limited to case records. The results of this study can only reflect what was recorded by staff and it is likely that some information failed to be recorded. This issue was addressed to some extent by the use of both computer and paper records, along with data routinely collected by the Trust's Mental Health Act Office. It is possible that this method underestimated both the degree of monitoring and the number of breaches that occurred.

### Characteristics of study sample

Patients on CTOs are typically males, around 40 years of age, with a long history of mental illness, previous admissions, with schizophrenia-like or serious affective illness. Our sample characteristics were consistent with these international findings.^2^

The characteristics of the sample are very similar to those seen in other parts of the UK,^5–8^ suggesting generalisability of results nationally.

### Main findings

A total of 179 category-specific conditions were identified in the sample. The average (modal) number of conditions per CTO was three. We recorded 95 breaches of additional conditions during the study period. Only eight patients were recalled following the breach of an additional condition, and seven of these had relapsed by the time they arrived in hospital.

A total of 25 patients were recalled during the study period, and 24 of the 25 had their CTO revoked and remained in hospital under Section 3 of the Mental Health Act because they had relapsed. This finding is consistent with the data from other years within the Trust: from November 2008 (when CTOs were first introduced) to February 2012 there were a total of 57 recalls and 56 revocations. Other reported data^9^ provide an overview of all uses of CTOs in England. From November 2008 to April 2011 there were a total of 10 071 new CTOs. Of these, there were 3025 recalls and 1940 CTOs were revoked. This suggests that on average 64% of patients recalled will then have their CTO revoked and remain in hospital on Section 3. This figure is lower than that found in the current study, but is still surprisingly high.

Both the revocation rate and the fact that breached conditions only rarely led to recall is interesting. We suggest that this might in part relate to how the law governing CTOs is interpreted. According to Section 17E(1) of the Mental Health Act, the responsible clinician may recall a community patient on a CTO to hospital if in their opinion:

- the patient requires medical treatment in hospital for their mental disorder, and
- there would be a risk of harm to the health or safety of the patient or other persons if the patient were not recalled to hospital for that purpose.

Therefore, only if the breach of the condition is associated with an increase in risk can the recall be justified. It is not clear whether the risk must be immediate to permit a lawful recall or whether a patient can be recalled based on the prediction that certain behaviours will lead to risk being present in the near future. The findings from our study suggest that clinicians interpret the risk of harm to be immediate. There are cases, however, where it is reasonable to predict that if the patient breaches a condition (for example, refuses their depot medication or misuses drugs), they will relapse relatively quickly and a risk of harm will soon follow that may not be mitigated by action from their clinical team. In our opinion, in such cases, the wording of Section 17E(1) permits clinicians to recall patients before they have relapsed based on the nature of their illness. Furthermore, if the risk stated in Section 17E(1) is taken to be immediate, then it follows that the patient must have demonstrated signs of relapse before recall, thus rendering CTOs ineffective at preventing relapse, one of their core aims.

In addition to the way in which Section 17E(1) is interpreted there are likely to be other reasons why clinicians wait until a patient has shown signs of relapse before recalling them. First, this has been the approach for many years and such a departure from routine practice may be too much of a challenge to the prevailing culture. Second, clinicians must balance the need to contain immediate risks with the need to preserve a therapeutic relationship with the patient, which is essential to manage risks in the longer term.

Despite the issues surrounding the recall of patients on CTOs, supervised community treatment has potential benefits. A cohort of patients appear to adhere to conditions out of concern that if they do not, they will be recalled, often resulting in improved mental health and reduced risk. Such behaviour was certainly observed in this study and it is reflected by experience in clinical practice. However, given that our study findings suggest that simply breaching conditions will not result in recall, it is arguable that such an approach, although bringing with it benefits for the patient, is overly paternalistic and is not in keeping with a culture of respecting human rights and being open with patients.

We recommend that clinicians should only include additional conditions that they would act on should the patient fail to comply. Such an approach will introduce clarity for both the patient and other members of the clinical team. It will also avoid the risk of the more important conditions being diluted by the patient's experience of breaching other conditions without consequence. Additionally, the inclusion of conditions that are unlikely to lead to recall could be considered an unnecessary restriction on the patient and such practice is inconsistent with the principles of the Mental Health Act's *Code of Practice*.^10^

### Clinical implications

Since their introduction in 2008, CTOs have become an increasingly common way to manage ‘revolving-door’ patients in the community. This is despite the lack of a clear evidence base to support their use. Additional conditions, which place restrictions on the patient, are considered an appropriate and necessary part of CTOs and are routinely included.

The high recall to relapse rate ratio recorded in this study suggests that responsible clinicians wait until patients show signs of relapse before recalling them. We suggest that this may occur for a number of reasons but most importantly it is due to the way in which the law governing CTOs is worded and interpreted. Our interpretation of the law is that CTOs do provide a legal mechanism which enables a patient to be recalled to hospital before they relapse, based on the nature of their illness.

The effectiveness of additional conditions, in part, relies on the patient's belief that if they breach conditions, they will be recalled to hospital. This does not appear to be borne out in practice, however, and additional conditions tend to contribute little to the decision to recall a patient on a CTO. Although it may improve a patient's mental health, to imply that the breach of a condition will result in recall when this is likely not to be the case is akin to providing a placebo and raises ethical issues.
